# Development of the preterm infant gut microbiome: a research priority

**DOI:** 10.1186/2049-2618-2-38

**Published:** 2014-10-13

**Authors:** Maureen W Groer, Angel A Luciano, Larry J Dishaw, Terri L Ashmeade, Elizabeth Miller, Jack A Gilbert

**Affiliations:** 1 University of South Florida College of Nursing, 12910 Bruce B. Downs Blvd., Tampa, FL 33612, USA; 2 Department of Pediatrics, Division of Neonatology, Morsani College of Medicine, University of South Florida, Tampa General Cir, Tampa, FL 33606, USA; 3 Department of Pediatrics, Morsani College of Medicine, ACH Children’s Research Institute, USF, 140 7th Avenue South, St. Petersburg, FL 33701, USA; 4 Department of Anthropology, University of South Florida, 4202 East Fowler Ave., Tampa, FL 33620, USA; 5 Institute for Genomics and Systems Biology, Argonne National Laboratory, Lemont, IL 60439, USA; 6 Department of Ecology and Evolution, University of Chicago, 5640 South Ellis Avenue, Chicago, IL 60637, USA; 7 College of Environmental and Resource Sciences, Zhejiang University, Hangzhou 310058, China

**Keywords:** Preterm infants, VLBW, Gut microbiota, Health

## Abstract

The very low birth weight (VLBW) infant is at great risk for marked dysbiosis of the gut microbiome due to multiple factors, including physiological immaturity and prenatal/postnatal influences that disrupt the development of a normal gut flora. However, little is known about the developmental succession of the microbiota in preterm infants as they grow and mature. This review provides a synthesis of our understanding of the normal development of the infant gut microbiome and contrasts this with dysbiotic development in the VLBW infant. The role of human milk in normal gut microbial development is emphasized, along with the role of the gut microbiome in immune development and gastroenteric health. Current research provides evidence that the gut microbiome interacts extensively with many physiological systems and metabolic processes in the developing infant. However, to the best of our knowledge, there are currently no studies prospectively mapping the gut microbiome of VLBW infants through early childhood. This knowledge gap must be filled to inform a healthcare system that can provide for the growth, health, and development of VLBW infants. The paper concludes with speculation about how the VLBW infants’ gut microbiome might function through host-microbe interactions to contribute to the sequelae of preterm birth, including its influence on growth, development, and general health of the infant host.

## Review

### Background

The variability of human life is extraordinary, yet this is not surprising considering the multitudinous influences wrought upon us by genetics, culture, and the environment. Human-associated microbial life, which is also highly diverse, is influenced by many of the same factors. The fact that microbial life provides considerable feedback on nearly all aspects of human physiology has not been widely appreciated. The rich diversity of microbial life represents an ancient evolutionary process of balance and benefit between host immunity and microbial growth [1]. The microbiota are so significant to health that NIH launched the Human Microbiome Project in 2007 [2] and, along with a diverse array of funding sources globally, continues to fund an extraordinary array of host-microbe investigations. Microbial life is known to develop in biological and anatomical niches and to be influenced by a host of ecological and genetic factors [3]. While microbes colonize every available surface in and on the body, the gut microbiome is most widely studied and consists of trillions of microbes and millions of functional genes, living in cooperation with each other and with the host [2]. The normal gut microbiota is estimated to consist of up to 100 trillion microorganisms, comprising between 500 to 3,000 species [4], and nearly 5 million unique genes, which is 100 times more genes than the human genome [5]. The human host requires this both benign and beneficial microbiota for normal function, including nutritional, developmental, defensive, and physiologic processes, resulting ultimately in immune tolerance to food and bacterial antigens, protection against pathogens, and maturation of the gastrointestinal epithelium [6]. The influence of the microbiome on host physiology and development is often considered to be immediate, but new evidence is emerging suggesting that changes in the microbiome early in life may affect host physiology across the life span (for an excellent synthesis of these concepts, we suggest *Missing Microbes* by Martin J. Blaser (NY: Henry Holt and Co., LLC, 2014)). As such, subtle shifts in the external factors that may influence the infant gut microbiome, including diet [7], must be properly catalogued if we are to be able to develop translational practices. Recent discoveries regarding the native microbiome in human milk indicate that this first food introduced into the gastrointestinal tract could have profound influences on how we modulate early gut microbiome development and, hence, the trajectory towards a stable, ‘adult’ microbiome [7].

#### ***Infancy and the gut microbiota***

Much of what is known about the infant microbiome has been from research on term infants, and fewer studies have focused on the preterm and low birth weight infants. Term and preterm infants were thought to be born essentially sterile, but new evidence of bacterial translocation in utero [8] and the presence of microbial life in preterm infants’ meconium [9] may refute this. Observed lipopolysaccharide (LPS) in the cord blood of preterm infants provides evidence of this translocation process [10]. While these findings are still controversial, they point to a profound ignorance of the founding of our microbiota. When born vaginally, infants are quickly colonized by trillions of maternal vaginal, enteric, skin, and milk microorganisms. The microbiota of neonatal skin, oral mucosa, and nasopharyngeal aspirates of vaginally delivered term infants are similar to their mother's vaginal microbiota, while Caesarean section (C-section) infants harbor bacterial communities similar to skin microbiota [11]. Over a relatively short period of time, the vaginally delivered newborn develops its first gut microbiome, which is largely composed of maternal vaginal and enteric organisms. Some data suggest that C-section-delivered infants have lower gut microbial richness and diversity at 4 months of age, compared to vaginally delivered infants [12]. Subsequently, during the first year of life in full-term infants, the gut is colonized by sequential blooms of microbes comprising approximately 10^14^ cells per milliliter of intestinal contents. Most are anaerobes in the distal small intestine and colon, but facultative anaerobes and aerobes also colonize the upper gastrointestinal tract. Because the lower gastrointestinal tract lumen is anoxic, only anaerobes and facultative bacteria are capable of surviving. The term infant’s gut microbiome matures over the first 3 years of life before it is recognizable as an adult microbiome [3,13].

The gut microbiota of the term infant has been observed to influence multiple processes including the differentiation of epithelium, cross talk within the gut-associated lymphoid tissue (GALT), gastrointestinal tract morphology, nutrition, and metabolism [14-18]. Human milk provides many nutrients for bacterial growth (including prebiotics such as human milk oligosaccharides (HMOs) [19]), and breast-fed infants have nearly twice the abundance of intestinal bacterial cells, but less diversity, than their formula-feeding counterparts (21). In term infants, the species of microbes that colonize the early gut differ between those who are breast-fed compared to formula-fed infants. HMOs are broken down into short-chain fatty acids that promote the growth of *Bifidobacteria* and *Lactobacillus*[20]. Formula-fed infant feces contain higher numbers of *Atopobium*, lower numbers of *Bifidobacterium*, and an increased relative abundance of *Bacteroides*[21]. Interestingly, when breast-fed babies receive mixed feeding of both human milk and formula, the microbiota shifts to a community structure that more closely resembles that found in formula-fed infants, which is similar to when the infants begin solid food [14]. This ‘formula/solid food’ microbiome is dominated by *Enterococci* and *Enterobacteria*, *Bacteroides*, *Clostridia*, and other anaerobic *Streptococci*[14]. Preterm infants may differ in the responsiveness of the gut to prebiotics such as HMOs. In one study, administration of supplemental HMOs to the feedings in 12 preterm infants did not result in the expected increase in *Bifidobacteria*[22]. Hence, manipulation of the preterm infant gut microbiome may be challenging because of different influences in the early lives of these infants compared to term infants. In general, there is much less known about the influence of human milk feeding on the gut microbiome of preterm infants, which represents a significant research gap.

Within the first year of life, the intestine will be colonized by approximately 1,000 different species [20]. While human milk contains complex nutrients, it also harbors many immune components and a recently described microbiome (10^3^ to 10^4^ colony-forming units of select species per milliliter of human milk) [23]. This symbiotic mix of probiotic and prebiotic components, combined with other sources including the environment and immune activation, acts to populate the early gut with a microbiota. Milk from preterm mothers is different in many ways from that produced by term mothers. For example, preterm milk appears to have a different mix of human milk oligosaccharides that could influence microbial growth [24].

The early gut microbiome is believed to maintain a state of regulated inflammation that is important in providing cross talk between microbes of the intestinal epithelium [6] and the GALT [20]. The development of a normal infant gut and the immune system requires interaction between intestinal epithelial cells, lymphoid tissue, and the burgeoning commensal microbiota. Apoptotic stimuli, reactive oxygen synthesis, and Toll-like receptor (TLR) signaling are induced by commensal bacteria to produce a state of controlled inflammation that helps develop the innate immune defenses and promotes pathogen recognition [25,26]. The developing commensal microbiota alters gut microanatomy and function. There is increased expression of genes that promote epithelial turnover and synthesis of mucus, as well as elevated peristalsis, which decreases small intestinal colonization; finally, various antimicrobial compounds are secreted in the mucus [27,28]. These interactions enable the infant gut to quickly develop tolerance to food antigens and the commensal microbiota. This provides a barrier against penetration of pathogenic microbes into the mucosa and submucosa.

After weaning, the gut microbiota becomes firmly established leading to a lifelong signature [29], a process that usually takes place by about 3 years of age [3]. However, the early microbiome is likely to determine the signature of the adult microbiome, based on the founder effect, whereby the original colonizing microbiota are instrumental in the successional direction of the assemblage. As a result, the initial colonizing microbiota is likely to be a critical determinant in the development of certain pediatric diseases [30]. The early microbiota produces active metabolites such as folate, butyrate, and acetate, which could epigenetically alter gut epithelium and hepatic and immune cells, a type of developmental programming which might later translate into risks for a variety of human diseases, including obesity [31].

#### ***The very low birth weight (VLBW) infant gut microbiome***

As shown above, the term infant gut microbiome has been the primary focus of existing research. Therefore, we highlight the need to refocus our research paradigm to fully explain how preterm birth might interrupt normal developmental pathways. VLBW infants (birth weight below 1,500 g) develop a very different, ‘sparse’ microbiome than that described for the full-term infant. This developmentally challenged microbiota is very low in anaerobes, with coagulase-negative *Staphylococci*, *Enterococci*, *Enterobacteriaceae*, and yeasts being common microbial species [27]. In addition, VLBW infants may be fed cow’s-milk-based formula or human milk fortifiers that can create an entirely different metabolic environment in the gut and thus may favor very different microbial communities [32]. Exposure to many prenatal and postnatal insults may shape the succession of the preterm infant gut microbiome. These infants often experience rapid vaginal or C-section deliveries reducing exposure to maternal vaginal and enteric microbiota. In addition, mothers threatening preterm delivery often experience prolonged hospitalization and are treated with antibiotics that may alter their microbiota, as well as that of the fetus. The infants can be exposed to many inflammatory factors in utero (prenatal maternal illness, infections such as bacterial vaginosis and chorioamnionitis, smoking and physiological stress) and then postnatally (formula feeding, invasive procedures, antibiotics and medications that alter gastroenteric pH) and extremely different microbial sources as compared to full-term neonates. Furthermore, the hospital environment is likely to be vastly different than home in terms of resident microbes and microbial resistance. VLBW infants require prolonged hospitalization and often develop a ‘neonatal intensive care unit (NICU)’ flora [33]. The VLBW infant gut is immature and fragile, and extended periods of fasting and alterations in gastrointestinal motility and perfusion may affect colonization and increase inflammation. It is logical that the composition of the VLBW infant gut microbial community will influence the successional development of the microbiota, and bacteria that established early on will inhabit important niches later in life. VLBW infants often display a delay in establishing an adult microbiota, compared to full-term children [20]. These delays and alterations in commensal colonization in early life may preempt gastroenteral illnesses and shifts in immune balance, leading to atopy and neurodevelopmental effects in childhood and later life [20].

One recent longitudinal study of two VLBW infants found that the gut microbiome was influenced by microorganisms in the NICU environment, and antibiotic-resistant microbes were colonizing these infants’ guts [33]. Genes conferring antibiotic resistance can transfer among microbes, including pathogens, a process termed the gut-associated resistome [34]. In another longitudinal study of a single VLBW infant, born by C-section, nine stool samples taken during the third week of life showed a shift towards fermentation-based metabolism (obligate anaerobes) which overtook *Escherichia coli* as the most abundant microbial species [35]. A very recent paper describes a study of microbial succession in 58 VLBW infants grouped into three gestational ages (<26 weeks, 26–28 weeks, and >28 weeks) [36]. Fecal samples (*N* = 922 specimens) were studied across these infant groups as the infants grew and developed. The results support a programmed and non-random developmental succession of microbiota, from *Bacilli* to *Gammaproteobacteria* to *Clostridia*, with colonization dominated by anaerobes by 33–36 weeks postconceptual age (weeks of age at birth + weeks of age since birth). There were frequent and abrupt unpredictable shifts in the composition of microbial life along the course of this trajectory. The pace of ecological succession, but not progression of taxonomic changes, was influenced by external factors such as breast milk feeding, antibiotics, and mode of birth. This important study will become even more valuable if the infants are subject to follow-up investigation later in life, so that the infant successional ecology and founder effect of their microbiome may be linked to early childhood illness progression and health status.

#### ***Effects of gut microbiome in early VLBW infants’ lives***

VLBW infant deaths contributed 35% to all infant deaths in 2009 (http://www.cdc.gov/reproductivehealth/MaternalInfantHealth/PretermBirth.htm). While most VLBW infants survive, the costs of care and long-term sequelae are highly significant. VLBW infants are at risk of hematological, cardiorespiratory, gastroenterologic, infectious, and neurological disorders in their early lives in the NICU (http://www.nlm.nih.gov/medlineplus/prematurebabies.html). These, as well as prenatal and birth-related insults, contribute to long-term morbidity. VLBW infants are usually the product of preterm birth (PTB), but some may be born closer to term (late preterm) and are small for gestational age. There are very few *prospective* studies of the VLBW gut microbiome over the NICU stay that assess more than a few VLBW infants [33-36].

The dysbiosis of microbial succession in VLBW infants is likely to increase the risk of infections and inflammatory processes. Sepsis is a major threat to these infants and may occur at any time during hospitalization. In a recent study, sepsis-causing pathogens were isolated from stools in 7 of 11 infants, and fecal and blood samples were provisionally matched. The organisms identified were not considered members of the normal microbiota (group B *Streptococcus*, *Serratia marcescens*, and invasive *E. coli*) [37]. Both late-onset sepsis and necrotizing enterocolitis (NEC) were associated with microbiomes dominated by *Proteobacteria* and *Firmicutes*[38]. NEC has an incidence of about 7% in the United States, and up to 10% of VLBW infants developed it [39,40]. Developmental immaturity in innate immune function may occur in VLBW infants, which interrupts the normal ability of enterocytes to control invasive non-commensal bacteria and results in a proinflammatory cascade [41]. Bacterial translocation across the epithelial barrier is associated with excessive TLR-4 signaling that produces the inflammation and necrosis characteristic of this disease [42]. What follows is an excessive inflammatory response that predisposes to NEC [20,41]. Use of human milk significantly reduces the risk for NEC and sepsis in the NICU [43], perhaps in part because human milk introduces a diversity of commensal microbial species and decreases the proinflammatory cascade that leads to NEC. Secretory immunoglobulin A (SIgA) in milk plays a role in immune exclusion by limiting the ability of microbes to penetrate into the epithelium, regulating commensal microbial growth, and affecting intestinal epithelial gene expression [44,45].

Few studies have mapped normal, non-medically treated full-term infants’ or VLBW infants’ microbiota over time after discharge from the hospital. Eggesbo et al. [46] describe the colonization process during the first 4 months of life in 85 healthy term infants not receiving medical interventions and demonstrated a developmental trajectory for the establishment of the adult-type microbiome. Normal early infancy colonization patterns showed less complexity and higher transient levels of facultative anaerobes in infancy compared to later in life [27]. The initial colonizers at 4 days of life were *Enterobacteriaceae*, with *Klebsiella* and *Enterobacter*, more common in neonatal gut compared to adults. *Bifidobacterium* species were also prevalent, as was *Lactobacilli. Bifidobacterium* is the dominant bacteria in the guts of breast-fed infants. At 4 months of age, dramatic differences in microbiota occurred, which likely was due to changes in feeding.

The adult-type microbiome, established by 3 years of age in healthy full-term newborns, is stable through childhood and into old age [30]. It is unlikely, however, that the same pattern and timing would occur in the VLBW infant; yet, there do not appear to be studies in the literature prospectively mapping the gut microbiome of VLBW infants through early childhood. This is an important gap as the role of the microbiome in the growth, health, and development of VLBW infants provides essential data to aid doctors in their care.

#### ***VLBW infant growth and the gut microbiome***

VLBW infants generally experience catch-up growth during early childhood, with most of this growth occurring in the first 2 years of life but continuing until 8 years of age [47]. This period of rapid growth and weight gain has been linked to later risk of coronary artery disease and insulin resistance [48]. One aspect of growth that may be related to the microbiome is the risk for later obesity (and attendant disorders such as metabolic syndrome and type 2 diabetes), particularly in small for gestational age infants. VLBW infants are often small for gestational age as well as prematurely born and could be more at risk for later obesity rather than underweight [49]. In addition, a preterm infant can suffer postnatal growth failure due to inadequate nutritional intake and illness, so that by term, they are considered ‘small for dates’. Known as the Barker or ‘thrifty phenotype’ hypothesis [50], the relationship between prenatal nutritional deprivation, either through maternal deprivation, high-altitude pregnancy, or placental insufficiency, is hypothesized to lead to a metabolic programming that ultimately increases the risk for later obesity in small for gestational age infants. Thus, later obesity has a U-shaped relationship with early nutrition, with low and high weight at birth increasing the risk for these metabolic alterations. General thinking has been that infants who have developed a metabolic adaptation to reduced prenatal nutrition are born with set points and mechanisms geared towards metabolic efficiency. This adaptive fetal phenotype may result in obesity when nutritional supply is no longer restricted after birth. The risk may be enhanced in VLBW small for gestational age infants due to the alterations described previously in the VLBW infants’ gut microbiome. Some studies suggest that there is an association between obesity development in VLBW infants and the rate of accelerated catch-up growth during infancy [49]. Another possibility is that normal nutrient digestion and processing, metabolism, and microbe-host signaling become disturbed in the VLBW infant due to dysbiosis, and this could contribute to obesity. More research is needed to explore this possible relationship over longer time spans.

The obesity-microbiome relationship has been the subject of many studies. In a very large study of 74,946 children from 31 centers in 18 countries, antibiotic treatment in the first 12 months of life was found to be associated with current BMI in boys, ages 5–8, but not girls [51]. In a study comparing obese to lean children, fecal samples were analyzed to determine the concentrations of bacterial species belonging to the following genera: *Bacteroides*, *Bifidobacterium*, *Clostridium*, *Staphylococcus*, *and Lactobacillus*[52]. There were significant differences in the microbiomes of obese compared to lean children (ages 6–16). The ratio of *Firmicutes* to *Bacteroidetes* was higher in obese children, and low levels of *Bacteroides vulgatus* and high concentrations of *Lactobacillus* spp*.* and *Staphylococcus* spp*.* were positively associated with energy intake in both obese and lean children. A relationship between inflammation (plasma high-sensitivity C-reactive protein [hsCRP]) and *Lactobacillus* spp*.* was observed in the obese children in this study. Many obese individuals (up to 50%), including children, develop insulin resistance and metabolic syndrome. In an adult study, fecal transplants from lean individuals to individuals with metabolic syndrome reversed the insulin resistance, implicating the microbiota in metabolic syndrome etiology [53]. Maternal prenatal BMI may influence the infant microbiome and later risk for obesity. The childhood obesity epidemic in the United States may be due to many factors, including diet and sedentary behaviors, but maternal influences may be important and may operate through the maternal prepregnancy and pregnancy BMI. Maternal obesity was associated with higher levels of *Bacteroides* and *Prevotella*, and these microorganisms appeared higher in the children at 1 and 6 months of age [54], suggesting intergenerational transfer of a microbiome that is associated with obesity [55].

#### ***VLBW infant development and the gut microbiome***

The VLBW infant is at risk for long-term disabilities, with increased risks for neurological sequelae including cognitive disabilities, blindness, cerebral palsy, and hearing impairments compared to infants of later gestational age [56]. The presence of attention deficit/hyperactivity disorder is five times higher in VLBWs compared to full-term infants [57]. In a 30-month follow-up of VLBW infants with gestational ages of 25 weeks or less, over half had developmental delays, neuromotor disability, blindness, or hearing loss [58]. While there are certainly clear relationships between gestational age, birth weight, and neurological impairment, the role of the microbiome has barely been investigated. There is an evolving literature, mostly with animal studies, showing the effect of the gut microbiome on neurogenesis and neuronal circuits involved in motor function, stress responses, and anxiety [59]. The gut-brain axis is both complex and extensive, with the enteric nervous system providing a mechanism for cross talk. Research has demonstrated that the microbiota is involved in this communication pathway (the ‘brain-gut-enteric-microbiota axis’) and may impact brain development and plasticity, as well as gene transcription and behavioral responses [60]. In adults, multiple studies have explored the relationship between the gut microbiome and a diverse number of neuropsychiatric illnesses: schizophrenia, mood disorders, autism spectrum disorders (ASDs), anorexia nervosa, Tourette syndrome, attention deficit hyperactivity disorder, narcolepsy, posttraumatic stress disorder, and chronic fatigue syndrome [61]. Anxiety also has been linked to the microbiome in mice, and brain plasticity has been speculated to be dependent on normal development of the microbiome [59]. It may be that pathogens leak from the gut under certain circumstances and the microbe-associated molecular patterns (MAMPs) react with TLRs to produce inflammatory signals that act on the brain. Another potential pathway may be through autoimmune mechanisms. These pathways have not been studied in VLBW neonate growth and development. It is possible that soft neurological signs and developmental delays in these children may be associated with microbial dysbiosis. One neurological relationship of abnormal infant microbial colonization is seen in ASD which is approximately three times more likely to develop in VLBW infants with gestational age <27 weeks compared with infants born at term. Studies show that the shorter the gestation, the greater the risk [62]. ASD has been consistently associated with evidence of both neuroinflammation and abnormal gut flora such as abnormal *Clostridia* species and skewed *Firmicutes* to *Bacteroidetes* ratios [63]. A relationship between gastrointestinal symptoms and autism severity has also been observed [64], pointing to a potential relationship with the gut microbiota. Importantly, several bacterial probiotic therapies have also been posited and have shown high efficacy in mouse models [65].

#### ***VLBW infant health and the gut microbiome***

The establishment of commensal microbiota influences the infant’s immune system development. In fact, there are remarkable differences in intestinal microbial interactions in infancy compared to adulthood. The early life gut responds very differently than the adult gut to inflammogenic stimuli such as the proinflammatory cytokine tumor necrosis factor-alpha (TNFα). The infant gut microbiome coevolves with the developing gut; in fact, the immature immune system and immune responses are primed by transient inflammatory signals from microbe-gut interactions [20,66]. The development of both the innate and acquired immune systems in infants requires orchestrated interactions and controlled inflammation within the developing gut. The intestinal immune system is very extensive, with up to 60% of the immune cells residing in the GALT [59]. Dendritic cells (DCs) sample the commensal microbes that are present in the gut lumen in a process that begins with recognition of the MAMPs by TLRs on intestinal epithelial cells and penetrating DCs. The TLR activation results in secretion of cytokines such as TSLP and TGF-β, both of which promote tolerogenesis in DCs [20]. DCs produce immunoregulatory cytokines such as IL-10 that results in upregulation of T regulatory cells. In addition, DCs transport bacteria to the mesenteric lymph nodes where B cells recognize epitopes and become IgA-secreting plasma cells specific to the microbes. This IgA is recirculated back to the lamina propria of the intestine, where it binds with a secretory piece and enters the intestinal lumen to contain bacteria from penetrating the epithelium [17]. By these mechanisms, the immune system protects against specific bacteria and isolates them to the gut. In the VLBW population, the enteric effects of abnormal colonization can interfere with these normal processes. There are potential developmental differences in intestinal epithelial cell responses, leading to decreased immunoregulation and increased inflammation, both of which may contribute to NEC. Another important mechanism is how the bacteria in the gut alter the systemic immune system, and these interactions offer explanation for the adverse health effects of inadequate or abnormal colonization. Depending on the microbial species, effects far distal to the gut can be observed, such as systemic autoimmune diseases and allergies, which appear to be influenced by immune balance orchestrated in the gut. If normal microbiota development is somehow impacted, as is likely the case for VLBW infants, there may be an increased risk for later enteric diseases and defects in immune tolerance, atopy, and asthma [20]. Immunological consequences of the abnormal microbiota profile that may occur in VLBW infants as they develop include disruption in the Th1/Th2/Th17/Treg immune balance, which may, in part, explain the relationship between inadequate colonization in VLBW infants and later atopic disease and asthma. Since VLBW infants are often born by C-section and have exposure to antibiotics in early life, they have additional risks. In a study of infants born by C-section with atopic family history, the odds ratio for development of asthma was 8 [67]. There is also a well-known epidemiological association between antibiotic use and later development of asthma, which often is explained by low microbial exposure (the hygiene hypothesis) [68,69]. Type I diabetes is an autoimmune-mediated metabolic disorder potentially associated with the microbiome. Newly diagnosed Mexican children with type I diabetes had higher fecal *Bacteroides* and reduced *Prevotella*, *Megamonas*, and *Acidaminococcus* compared to control children. Diabetic children also had higher antibiotic exposure than controls [70].

Enteric effects of the VLBW microbiome are not limited to the early postnatal period. Antibiotic exposure, which is an extremely common intervention in VLBWs, has been associated with increased later risk for Crohn’s disease, with increasing risk related to frequency of antibiotic exposure in children [71]. In another study, C-section delivery and being small for gestational age increased the risk of celiac disease [67]. Obviously, these factors may or may not be associated with the microbiome.

## Conclusions

It is becoming increasingly clear that the early infant gut microbiome is an important regulator of many developing physiologies. These interactions can influence risk for a variety of pathophysiological effects into childhood and even adult life. It is also apparent that infant feeding is a critical determinant of the early gut microbiome, and so, early feeding habits may have important long-term effects. The VLBW infant is disadvantaged in many ways, including the likely development of dysbiosis of the gut microbiome. Exploration of the long-term effects of this abnormal microbial number, diversity, and succession has not yet been done and is critically needed in order to intervene both early and later in the lives of these children.

## Abbreviations

ASD: autism spectrum disorder; C-section: Caesarean section; DCs: dendritic cells; GALT: gut-associated lymphoid tissue; hsCRP: high-sensitivity C-reactive protein; LPS: lipopolysaccharide; MAMPs: microbe-associated molecular patterns; NEC: necrotizing enterocolitis; NICU: neonatal intensive care unit; PTB: preterm birth; SIgA: secretory immunoglobulin A; TLRs: Toll-like receptors; VLBW: very low birth weight.

## Competing interests

The authors declare that they have no competing interests.

## Authors’ contributions

MWG developed the first draft of this review. AAL, TLA, EM, and LJD provided critical reviews and updated new references and suggestions for the content. JAG provided intellectual oversight, suggestions for expansion and new direction, multiple critiques, and final editing. All authors read and approved the final manuscript.
